# Frequency of *Anaplasma phagocytophilum*, *Borrelia* spp., and coinfections in *Ixodes ricinus* ticks collected from dogs and cats in Germany

**DOI:** 10.1186/s13071-024-06193-w

**Published:** 2024-02-23

**Authors:** Julia Probst, Andrea Springer, Volker Fingerle, Christina Strube

**Affiliations:** 1 Institute for Parasitology, Centre for Infection Medicine, University of Veterinary Medicine Hannover, Buenteweg 17, 30559 Hanover, Germany; 2 National Reference Centre for Borrelia, Bavarian Health and Food Safety Authority, Veterinärstraße 2, 85764 Oberschleissheim, Germany

**Keywords:** Castor bean tick, Engorgement, Tick-borne diseases, Borreliosis, Anaplasmosis, Prevalence

## Abstract

**Background:**

Changing geographical and seasonal activity patterns of ticks may increase the risk of tick infestation and tick-borne pathogen (TBP) transmission for both humans and animals.

**Methods:**

To estimate TBP exposure of dogs and cats, 3000 female *I. ricinus* from these hosts were investigated for *Anaplasma phagocytophilum* and *Borrelia* species.

**Results:**

qPCR inhibition, which was observed for ticks of all engorgement stages but not questing ticks, was eliminated at a template volume of 2 µl. In ticks from dogs, *A. phagocytophilum* and *Borrelia* spp. prevalence amounted to 19.0% (285/1500) and 28.5% (427/1500), respectively, while ticks from cats showed significantly higher values of 30.9% (464/1500) and 55.1% (827/1500). Accordingly, the coinfection rate with both *A. phagocytophilum* and *Borrelia* spp. was significantly higher in ticks from cats (17.5%, 262/1500) than dogs (6.9%, 104/1500). *Borrelia* prevalence significantly decreased with increasing engorgement duration in ticks from both host species, whereas *A. phagocytophilum* prevalence decreased only in ticks from dogs. While *A. phagocytophilum* copy numbers in positive ticks did not change significantly over the time of engorgement, those of *Borrelia* decreased initially in dog ticks. In ticks from cats, copy numbers of neither *A. phagocytophilum* nor *Borrelia* spp. were affected by engorgement. *Borrelia* species differentiation was successful in 29.1% (365/1254) of qPCR-positive ticks. The most frequently detected species in ticks from dogs were *B. afzelii* (39.3% of successfully differentiated infections; 70/178), *B. miyamotoi* (16.3%; 29/178), and *B. valaisiana* (15.7%; 28/178), while *B. afzelii* (40.1%; 91/227), *B. spielmanii* (21.6%; 49/227), and *B. miyamotoi* (14.1%; 32/227) occurred most frequently in ticks from cats.

**Conclusions:**

The differences in pathogen prevalence and *Borrelia* species distribution between ticks collected from dogs and cats may result from differences in habitat overlap with TBP reservoir hosts. The declining prevalence of *A. phagocytophilum* with increasing engorgement duration, without a decrease in copy numbers, could indicate transmission to dogs over the time of attachment. The fact that this was not observed in ticks from cats may indicate less efficient transmission. In conclusion, the high prevalence of *A. phagocytophilum* and *Borrelia* spp. in ticks collected from dogs and cats underlines the need for effective acaricide tick control to protect both animals and humans from associated health risks.

**Graphical Abstract:**

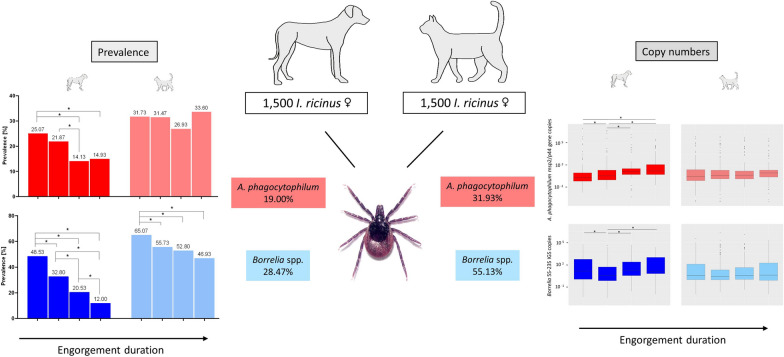

## Background

Recent changes in geographical distribution and seasonal activity patterns of ticks, such as the castor bean tick *Ixodes ricinus*, may increase the risk of tick infestation and tick-borne pathogen (TBP) transmission for both humans and animals. While in humans Lyme borreliosis caused by species of the *Borrelia burgdorferi* sensu lato (s.l.) complex is the most frequent tick-borne disease (TBD) in Europe, borreliosis in dogs is less common but may be associated with febrile illness, polyarthritis, and glomerulonephritis [1]. Next to species of the *B. burgdorferi* s.l. complex, *B. miyamotoi* causing relapsing-fever-like illness is frequently detected in hard ticks from Germany [2, 3]. Moreover, granulocytic anaplasmosis caused by *Anaplasma phagocytophilum* is common in dogs [4, 5] with increasing incidence and clinical relevance in Germany [6]. In severe cases, the Gram-negative and obligate intracellular bacterium can lead to severe febrile, life-threatening illness, sometimes accompanied by central nervous system disorders or gastrointestinal symptoms [7, 8]. Cats may also be affected, and clinical cases of *A. phagocytophilum*-positive felines with lethargy, loss of appetite, fever, severe inflammatory processes, and thrombocytopenia are increasingly reported [5, 9, 10].

Several European studies have examined the prevalence of *A. phagocytophilum* in field-collected questing ticks, resulting in values between 1.0% in a study from Germany [11] and 40.5% in a study from Denmark [12]. Prevalence is typically higher in adult than nymphal ticks, as shown for example in a study from Denmark with infection rates of 14.5% in nymphs compared with 40.5% in adult *I. ricinus* ticks [12]. Local investigations in the northern German cities of Hamburg and Hannover revealed *A. phagocytophilum* prevalences of 2.1% and 4.5%, respectively [13, 14], in field-collected adult *I. ricinus*, compared with only 1.0% in a study from southern Germany [11]. In ticks collected from dogs and cats in Europe, between 1.0% and 22.3% were qPCR-positive for *A. phagocytophilum*, with most of the studies concentrating mainly on dogs [15–20].

Similarly, *Borrelia* prevalence in questing ticks varies considerably between different regions and countries in Europe, with a mean prevalence of 3.6% in the British Isles to 19.3% in Central Europe [21]. Similar to *A. phagocytophilum*, the average infection rate is higher in *I. ricinus* adults (14.9%) than in nymphs (11.8%) [21]. A study from the City of Hanover, northern Germany, monitored the *Borrelia* spp. prevalence at the same locations over several years, showing a rather stable prevalence between 22.7% and 25.5%, with significantly higher infection rates in adult ticks than in nymphs, e.g., 32.6% versus 18.4% in 2020 [22]. In another study from northern Germany, a prevalence of 31.7% in adults and 28.6% in nymphs was detected [3]. Although the clinical relevance of Lyme borreliosis in dogs may be rather low [1] and questionable in cats [23], assessing the prevalence of *Borrelia* spp. in ticks from dogs and cats does not only shed light on their own infection risk but may also serve as an indicator of human risk. Dogs usually accompany their owners, and in addition, both dogs and cats may introduce ticks into the home, which could represent a human health risk. In ticks collected from dogs, the overall *Borrelia* prevalence varied between 2.1% in the UK and 44.0% in the Ukraine [24, 25]. For ticks from cats, one study reported a *Borrelia* prevalence of 18.0% [26], while another study indicated a prevalence of 10.2% for ticks from both host species [27].

The use of ticks removed from hosts for prevalence studies is not without controversy, as ticks detached from a host may have acquired the infection during the actual bloodmeal or by cofeeding transmission [28]. Nevertheless, the main aim of the recent study was to assess the TBP contact risk for dogs and cats and to unravel potential host-related differences. Moreover, taking tick engorgement status into account may help to clarify the direction of pathogen transmission during the bloodmeal (“tick to host” or “host to tick”) on the basis of different patterns of prevalence between non-engorged and engorged ticks. A study from northeastern Poland showed differences in pathogen prevalence between the stages of engorgement of ticks collected from dogs [17]. While 34.4% of 436 engorged ticks were positive for *Borrelia* spp., only 4.7% of 21 non-engorged ticks were positive. This increased prevalence in engorged ticks is in contrast to a publication from southern Germany, which reported a prevalence of 33.3% in 26 non-engorged and 13.6% in 110 engorged female *I. ricinus* [29].

The wide range in reported prevalence values indicates the need for a comprehensive study on ticks collected from dogs and cats to assess their actual infection risk. Therefore, the present study investigated 3000 female *I. ricinus* specimens removed from dogs and cats during a Germany-wide tick collection study [30]. Ticks were selected to equally represent the two host species and different stages of engorgement to allow a meaningful comparison of *Borrelia* spp. and *A. phagocytophilum* prevalence and infection intensity in terms of qPCR copy numbers. To exclude interference of engorgement or bloodmeal, respectively, with qPCR sensitivity, a spiking experiment was performed and the PCR setup was adjusted accordingly.

## Methods

### Tick collection study and sample selection

In the frame of a tick collection study, 197 veterinary practices distributed across Germany submitted ticks between March 2020 and October 2021 [30]. Information on the clinical presentation of sampled hosts was not available. The submitted specimens were morphologically identified, and female *I. ricinus* specimens were measured using the OLYMPUS cellSens Entry (v. 3.2) software paired with an OLYMPUS SC50 camera adapter to determine the coxal index as an estimate of attachment duration [30, 31]. On this basis, female *I. ricinus* were assigned to one of four engorgement categories (non-engorged [0 h to ≤ 24 h], partially engorged stage one [> 24 h to ≤ 72 h], partially engorged stage two [> 72 h to ≤ 144 h], fully engorged [> 144 h]), which were adapted from Franta et al. [32] by dividing the period of “slow feeding” into two stages for a better differentiation within this long time span (> 24 h to ≤ 144 h). Ticks with implausible calculated engorgement times > 245 h were excluded. The ticks were then stored individually at −20 °C until further use.

**Fig. 1:**
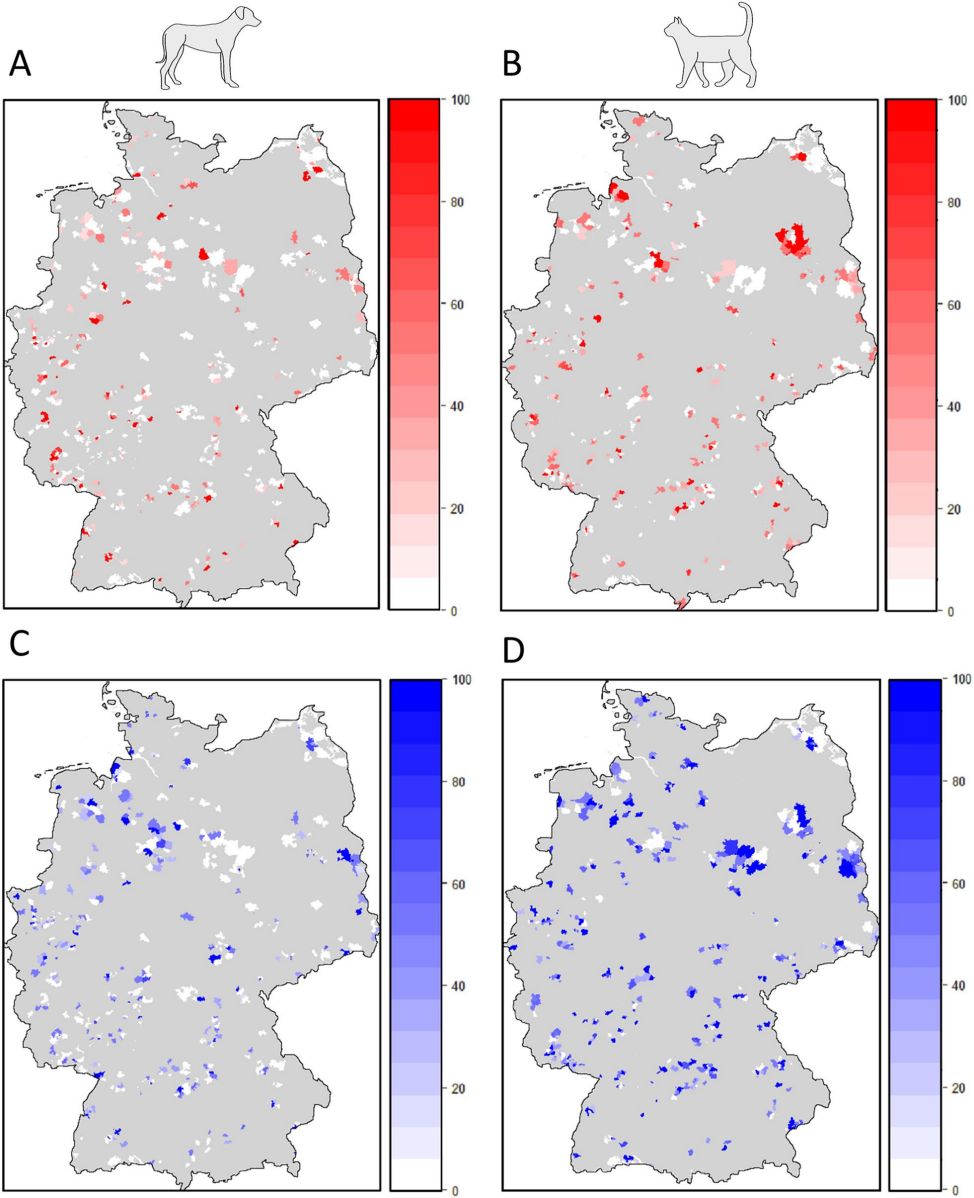
Nationwide distribution pattern of ticks positive for *A. phagocytophilum* (shades of red) and *Borrelia* spp. (shades of blue) from dogs (**A**/**C**) and cats (**B**/**D**). The shading indicates the proportion of positive ticks per sampled zip code. Zip code areas that were not sampled are shown in gray

From the total number of 14,383 received *I. ricinus* specimens, a representative sample of 3000 females, 1500 from dogs and 1500 from cats, was selected for pathogen analyses. Per host species, 375 specimens of each engorgement category (non-engorged, partially engorged stage one, partially engorged stage two, and fully engorged) were included. Within these categories, further selection according to the coxal index was performed to represent the stages of engorgement as evenly as possible. Additionally, ticks from dogs and cats infested with a single or multiple ticks (hereafter single and multiple infestations) were selected approximately equally as well, with 1–5 ticks analyzed per multiple infested animal. Within these categories, the samples were further chosen via randomized numbers. The origin of the selected samples was plotted on a map of Germany using R version 4.1.0 [33] with geographic data distributed by OpenStreetMap under the Open Database License (www.openstreetmap.org/copyright) to exclude a geographic bias (Fig. 1).

### Genomic DNA isolation

Ticks were homogenized in 20 µl phosphate buffered saline with polystyrene pistils (Carl Roth GmbH, Karlsruhe, Germany) in individual tubes, before addition of 25 µl proteinase K and 200 µl BQ1 buffer from the Nucleospin^®^ 96 Blood Core Kit (Macherey–Nagel GmbH & Co KG, Düren, Germany) and overnight incubation at 56 °C. Further, the DNA isolation followed the instructions of the Nucleospin^®^ 96 Blood Core Kit, with minor modifications as described [14, 34, 35]. The 100 µl eluate of genomic DNA was stored at −20 °C until further use.

### Detection of *A. phagocytophilum* and *Borrelia* spp. by quantitative real-time PCR evaluated by spiking experiments

The success of the DNA isolation was checked randomly for 100 ticks each from dogs and cats by targeting the *I. ricinus* internal transcribed spacer (ITS) 2 region in probe-based qPCR [36], including a serial plasmid standard (10^0^–10^6^ copies per reaction, ligated into TOPO^™^ TA vectors [Invitrogen™, Thermo Fisher Scientific, Schwerte, Germany]) and a negative control. Because of the high concentration of tick DNA from engorged specimens, which may inhibit exponential PCR amplification [37], 1 µl tick DNA template was used in the 25 µl reaction volume for ITS2 detection. The Mx3005 multiplex quantitative polymerase chain reaction (qPCR) system (Agilent Technologies Inc., Santa Clara, USA) was used for all qPCR reactions. The reaction setup has been described in detail by Strube et al. [36] with previously published adaptations by May and Strube [13]. The thermoprofile for both reactions included initial denaturation at 95 °C for 15 min followed by 45 cycles of 95 °C for 15 s and 60 °C for 60 s.

For pathogen detection, a duplex probe-based qPCR was performed targeting the *msp2/p44* multi copy gene of *A. phagocytophilum* [38] and the 5S-23S rRNA intergenic spacer (IGS) region [36], which occurs in duplicate in the *B. burgdorferi* s.l. complex and as a single copy in *B. miyamotoi* [39]. The qPCR setup included a negative control and serial plasmid standards (10^0^–10^6^ copies per reaction, ligated into TOPO™ TA vectors [Invitrogen™, Thermo Fisher Scientific, Schwerte, Germany]) for quantification. All samples were tested in duplicate. Initially, 10 µl template was used in the 25 µl reaction volume, while negative samples were later retested using only 2 µl on the basis of the result of a spiking experiment performed to exclude possible false-negative results due to PCR inhibition in engorged ticks. The spiking experiment was performed with six samples from each engorgement category and each host species (*n* = 48), which showed a negative qPCR result based on 10 µl template. Of these samples, 10, 5, and 2 µl template were tested as described above (if enough template was left), using a mastermix spiked with the *Borrelia* spp. and *A. phagocytophilum* plasmid standard, amounting to 10^4^ plasmid copies each per qPCR reaction. Further, a positive control containing only the plasmid standards but no template was included to generate reference cycle threshold (Ct) values. A second spiking experiment was conducted using 10 µl and 2 µl DNA of 22 non-engorged laboratory-bred *I. ricinus* females as template.

### *Borrelia* species identification by reverse line blot (RLB)

For all *Borrelia* qPCR-positive ticks, a conventional PCR targeting the 5S-23S ribosomal IGS region with primers B5S-Bor, BMiya-For, and 23S-Bor was performed [40, 41], followed by RLB differentiation with 11 different *Borrelia* species-specific probes as described before [40, 42]. Extrapolated from the spiking experiments (see section above), the amount of template was reduced from 10 µl to 2 µl for samples that were qPCR-positive with 2 µl but not 10 µl template volume, to avoid potential interference and inhibition in the RLB as well. The following *Borrelia* strains were used as positive controls: PAbe (*B. burgdorferi* sensu stricto (s.s.), PWudII (*B. garinii*), PBas (*B. afzelii*), VS116 (*B. valaisiana*), PHap (*B. spielmanii*), PBi (*B. bavariensis*), Poti B2 (*B. lusitaniae*), SCW-22^T^ (*B. carolinensis*), DN127 (*B. bissettiae*), and 25,015 (*B. kurtenbachii*), HT31 (*B. miyamotoi*). Owing to cross-reactions of *B. garinii* and *B. bavariensis* as well as *B. burgdorferi* s.s. and *B. carolinensis*, respectively, PCR products reacting with probes GA and SS were reamplified and custom Sanger sequenced (Microsynth Seqlab GmbH, Göttingen, Germany).

### Statistical analyses

Statistical analyses were performed using R version 4.1.1717 [33]. The *A. phagocytophilum* and *Borrelia* spp. prevalence, the coinfection rate, and the number of mixed infections with different *Borrelia* species was compared between ticks from dogs and cats via *χ*^2^ test or Fisher’s exact test in case of small values.

Further, generalized linear mixed models (GLMM) with binomial error structure were constructed separately for ticks from dogs and cats to analyze the influence of different predictors on the *A. phagocytophilum* and *Borrelia* spp. prevalence. As fixed factors, the tick’s stage of engorgement, whether the tick was from a single or a multiple infestation, and its coinfection status with *A. phagocytophilum* or *Borrelia* spp., respectively, were included in the model, while host animal ID was entered as a random factor. Multiple comparisons between the different stages of engorgement were performed using the function “glht” from the package “multcomp” [43] with Tukey contrasts. The full models were compared with null models based only on the random factor using likelihood ratio tests (R function “anova”).

The detected copy numbers for the msp2/p44 gene of *A. phagocytophilum* and the 5S-23S rRNA IGS region of *Borrelia* spp. (in the following referred to as *A. phagocytophilum* and *Borrelia* spp. copy numbers) were compared among the different engorgement categories of ticks from dogs and cats, respectively, via Kruskal–Wallis rank-sum tests followed by Dunn’s all-pairs rank-comparison test in case of a significant result. For all samples, the obtained copy numbers were extrapolated to 100 µl to quantify the copy number per examined tick.

Finally, the overall *Borrelia* species distribution was compared between the different engorgement categories of ticks from dogs and cats, respectively, by Fisher’s exact tests with simulated *P*-values. In case of a significant result, the prevalence of each species was compared between the engorgement categories by individual Fisher’s exact tests, followed by Bonferroni–Holm correction of *P*-values.

## Results

### Spiking experiments to evaluate pathogen qPCR detection

Using 10 µl template, 35.4% (17/48) and 29.2% (14/48) of the spiked tick DNA samples from dogs and cats tested negative for *A. phagocytophilum* and *Borrelia* spp., respectively, with no differences in the detection rate between ticks from dogs and cats. False negatives occurred independently of the engorgement duration. Further, Ct values were unaffected by the engorgement state. With decreasing amount of template, the number of false-negative results decreased, while Ct values of positive samples stayed mostly consistent, indicating that qPCR inhibition was not gradual but occurred in a “yes or no” manner. With 5 µl template, detection of *A. phagocytophilum* was still not successful in 2.1% (1/48) of all tick samples, while 18.8% (9/48) of the tick samples still yielded a false-negative *Borrelia* result. With 2 µl template, in all tick samples from which template was left (40/48; 83.3%), no PCR inhibition was noted for *A. phagocytophilum* nor *Borrelia* species.

When using DNA from unfed laboratory-bred ticks, no difference between 10 µl or 2 µl template was noted, as in both spiking experiments the detection rate was 100% (22/22) with Ct values consistent with the positive control.

### Pathogen prevalence in ticks collected from dogs and cats

Detection of the *I. ricinus* ITS2 region indicated successful DNA isolation of all randomly selected samples. However, as the spiking experiment revealed inhibition of the qPCR when using 10 and 5 µl template of ticks detached from dogs and cats independent of engorgement status, all tick samples initially negative using 10 µl were retested with 2 µl template only.

Of the analyzed ticks collected from dogs, 19.0% (285/1500) were infected with *A. phagocytophilum* and 28.5% (427/1500) with *Borrelia* spp., with a coinfection rate of 6.9% (104/1500). Detected copy numbers were mainly > 10^1^ but ≤ 10^2^, precisely for 42.5% (121/285) of *A. phagocytophilum* and 32.1% (137/427) of *Borrelia* spp. positive samples, with an overall median of 6.88 × 10^1^ and 1.07 × 10^2^ copies, respectively (Table 1).

**Table 1:** *Anaplasma phagocytophilum* and *Borrelia* spp. copy number distribution among positive female *I. ricinus* collected from dogs and cats

Copy number	Ticks from dogs		Ticks from cats	
	*A. phagocytophilum*	*Borrelia* spp.	*A. phagocytophilum*	*Borrelia* spp.
< 10^1^	15/285 (5.3%)	49/427 (11.5%)	31/464 (6.7%)	95/827 (11.5%)
≥ 10^1^ < 10^2^	121/285 (42.5%)	137/427 (32.1%)	177/464 (38.2%)	335/827 (40.5%)
≥ 10^2^ < 10^3^	90/285 (31.6%)	79/427 (18.5%)	173/464 (37.3%)	164/827 (19.8%)
≥ 10^3^ < 10^4^	17/285 (6.0%)	59/427 (13.8%)	24/464 (5.2%)	89/827 (10.8%)
≥ 10^4^	42/285 (14.7%)	103/427 (24.1%)	59/464 (12.7%)	144/827 (17.4%)
Median	6.88 × 10^1^	1.07 × 10^2^	1.17 × 10^2^	9.00 × 10^1^

Regarding ticks from cats, significantly more specimens were qPCR-positive compared with dogs, with 30.9% (464/1500) samples positive for *A. phagocytophilum* (*χ*^2^ = 56.377, *df* = 1, *P* < 0.001) and 55.1% (827/1500) for *Borrelia* spp. (*χ*^2^ = 218.13, *df* = 1, *P* < 0.001). Further, the coinfection rate of 17.5% (262/1500) was significantly higher in ticks from cats than from dogs (*χ*^2^ = 76.705, *df* = 1, *P* < 0.001). Like in ticks from dogs, copy numbers were comparably low, with 38.2% (177/464) of the samples containing > 10^1^ but ≤ 10^2^ copies for *A. phagocytophilum* and 40.5% (335/827) for *Borrelia* spp., with an overall median of 1.17 × 10^2^ and 9.00 × 10^1^ copies, respectively (Table 1).

Further, the GLMM revealed a significant positive association of *A. phagocytophilum* and *Borrelia* spp. in ticks from dogs, but not in those from cats (Table 2). There was no distinct geographical pattern in prevalence, with infected ticks originating from all over the country (Fig. 1).

**Table 2:** Results of the GLMM testing the influence of several predictor variables on the prevalence of *A. phagocytophilum* (model A and B) and *Borrelia* spp. (model C and D) infections in female *I. ricinus* collected from dogs and cats. The models were significantly different from a null model containing only the animal identification number as a random factor (A: chi-square = 26.36, *df* = 5, P < 0.001; B: chi-square = 15.14, *df* = 5, *P* = 0.01; C: chi-square = 145.64, *df* = 5, *P* < 0.001; D: chi-square = 30.63; *df* = 5, *P* < 0.001). Significant *P*-values are shown in bold

*A. phagocytophilum*	Model A: dogs				Model B: cats			
	Estimate	SE	*z*	*P*	Estimate	SE	*z*	*P*
Intercept	−2.00	0.42	−4.725	** < 0.001**	−1.35	0.20	−6.682	** < 0.001**
Stage of engorgement^a^								
Non-engorged versus partially engorged stage 1	−0.23	0.24	−0.977	0.760	−0.03	0.18	−0.144	0.999
Non-engorged versus partially engorged stage 2	−1.01	0.29	−3.429	**0.003**	−0.24	0.18	−1.303	0.561
Non-engorged versus fully engorged	−0.84	0.30	−2.851	**0.022**	0.13	0.18	0.74	0.881
Partially engorged stage 1 versus partially engorged stage 2	−0.78	0.28	−2.748	**0.030**	−0.21	0.18	−1.162	0.651
Fully engorged versus partially engorged stage 1	−0.61	0.28	−2.173	0.129	0.16	0.18	0.882	0.814
Fully engorged versus partially engorged stage 2	0.16	0.28	0.576	0.939	0.37	0.18	2.032	0.176
Infestation type (ref: single)								
Multiple	0.20	0.21	0.936	0.349	0.53	0.16	3.260	**0.001**
Co-infection with *Borrelia* spp.	0.38	0.20	1.860	0.063	0.07	0.13	0.515	0.606

*Borrelia* spp.	Model C: dogs				Model D: cats			
	Estimate	SE	*z*	*P*	Estimate	SE	*z*	*P*
Intercept	−0.23	0.15	−1.536	0.125	0.45	0.15	2.923	**0.003**
Stage of engorgement^a^								
Non-engorged versus partially engorged stage 1	−0.65	0.16	−4.185	** < 0.001**	−0.43	0.16	−2.647	**0.040**
Non-engorged versus partially engorged stage 2	−1.30	0.17	−7.461	** < 0.001**	−0.55	0.16	−3.425	**0.003**
Non-engorged versus fully engorged	−1.90	0.21	−9.256	** < 0.001**	−0.78	0.16	−4.781	** < 0.001**
Partially engorged stage 1 versus partially engorged stage 2	−0.64	0.17	−3.678	**0.001**	−0.12	0.16	−0.788	0.860
Fully engorged versus partially engorged stage 1	−1.25	0.20	−6.184	** < 0.001**	−0.35	0.16	−2.195	0.125
Fully engorged versus partially engorged stage 2	−0.61	0.21	-2.843	**0.023**	−0.23	0.16	−1.441	0.474
Infestation type (ref: single)								
Multiple	0.12	0.14	0.910	0.363	0.27	0.13	2.018	**0.044**
Co-infection with *A. phagocytophilum*	0.31	0.15	2.038	**0.042**	0.06	0.12	0.475	0.635

*SE * standard error, *GLMM*  generalized linear mixed model

^a^Multiple comparisons were conducted using the function “glht” from the R package “multcomp” [42] with Tukey contrasts

### *Borrelia* species identification by reverse line blot (RLB)

Regarding the differentiation of *Borrelia* species, the RLB was successful in 29.2% (366/1254) of the qPCR-positive ticks from dogs and cats, whereby the differentiation success depended on the number of 5S-23S IGS copies detected. The highest identification success of 70.9% (175/247) was achieved in samples with ≥ 10^4^ copies, followed by 53.4% (79/148) in samples with ≥ 10^3^ and < 10^4^ copies, 18.9% (46/243) in samples with ≥ 10^2^ and < 10^3^ copies, 10.8% (51/472) in samples with ≥ 10^1^ and < 10^2^ copies, and 10.4% (15/144) in samples with < 10 copies.

In successfully differentiated samples from dogs, the most frequently detected *Borrelia* species was *B. afzelii* (70/161; 43.5%), followed by *B. miyamotoi* (29/161; 18.0%), *B. valaisiana* (28/161; 17.4%), *B. garinii*/*B. bavariensis* (23/161; 13.7%), *B. spielmanii* (14/161; 8.7%), *B. burgdorferi* s.s./*B. carolinensis* (13/161; 8.1%), and *B. lusitaniae* (2/161; 1.2%) (Table 3). Coinfections were detected in 18 ticks (Table 4). Sanger sequencing of the 22 *B. garinii*/*B. bavariensis*-positive ticks revealed 21 (95.5%) as *B. garinii*, while no further differentiation was possible for one tick owing to a coinfection (Table 4). Further, *B. burgdorferi* s.s. was identified in 11/13 (76.9%) *B. burgdorferi* s.s./*B. carolinensis*-positive ticks, while sequencing failed for the three remaining samples (23.1%) owing to coinfections.

**Table 3:** *Borrelia* species distribution among qPCR-positive female *I. ricinus* from dogs and cats. Significant *P*-values (Bonferroni-corrected) are shown in bold

*Borrelia* species	Ticks from dogs %	Ticks from cats %	*χ*^2^	*df*	*P*-value	Performed test
*B. afzelii*	70/161 (43.5)	91/204 (44.6)	0.01	1	0.973	Chi-square test
*B. garinii*/*B. bavariensis*	22/161 (13.7)	19/204 (9.3)	1.30	1	0.254	Chi-square test
*B. burgdorferi* s.s./ *B. carolinensis*	13/161 (8.1)	10/204 (4.9)	1.04	1	0.307	Chi-square test
*B. bissettiae*	0/161 (0.0)	0/204 (0.0)				
*B. kurtenbachii*	0/161 (0.0)	0/204 (0.0)				
*B. lusitaniae*	2/161 (1.2)	6/204 (2.9)	n.a	n.a	0.416	Fisher’s exact test
*B. miyamotoi*	29/161 (18.0)	32/204 (15.7)	0.20	1	0.653	Chi-square test
*B. spielmanii*	14/161 (8.7)	49/204 (24.0)	13.74	1	** < 0.001**	Chi-square test
*B. valaisiana*	28/161 (17.4)	20/204 (9.8)	3.90	1	**0.048**	Chi-square test

*n.a.* not applicable for Fisher’s exact test

**Table 4:** Coinfections with different *Borrelia* species in female *I. ricinus* specimens collected from dogs and cats

Coinfection	Ticks from dogs	Ticks from cats
*Baf* + *Bmi*	–	5 (21.7%)
*Baf* + *Bsp*	2 (11.1%)	6 (26.1%)
*Baf* + *Bss/Bca*	6 (33.3%)	3 (13.0%)
*Bga/Bba* + *Bmi*	2 (11.1%)	1 (4.4%)
*Bga/Bba* + *Bsp*	–	1 (4.4%)
*Bga/Bba* + *Bss/Bca*	1 (5.6%)	–
*Bga/Bba* + *Bva*	2 (11.1%)	3 (13.0%)
*Bsp* + *Bmi*	2 (11.1%)	1 (4.4%)
*Bsp* + *Bva*	1 (5.6%)	1 (4.4%)
*Bss/Bca* + *Bmi*	1 (5.6%)	1 (4.4%)
*Bva* + *Bmi*	1 (5.6%)	1 (4.4%)
Total	18	23

*Baf* = *B. afzelii*; *Bga/Bba* = *B. garinii*/*B. bavariensis*; *Bmi *= *B. miyamotoi*; *Bsp* = *B. spielmanii*; *Bss/Bca* = *B. burgdorferi s.s.*/*B. carolinensis*; *Bva* = *B. valaisiana*

As in ticks from dogs, *B. afzelii* was the most frequently detected species in ticks from cats (91/204; 44.6%). Further, *B. spielmanii* (49/204; 24.0%), *B. miyamotoi* (32/204; 15.7%), *B. valaisiana* (20/204; 9.8%), *B. garinii*/*B. bavariensis* (19/204; 9.3%), and *B. burgdorferi* s.s./*B. carolinensis* (10/204; 4.9%) were detected, as well as *B. lusitaniae* (6/204; 2.9%), which was not detected in ticks from dogs. The *Borrelia* species distribution differed significantly as compared with ticks from dogs, with significantly more frequent detection of *B. spielmanii* but less frequent detection of *B. valaisiana* (Table 3). Coinfections were noted in 23 ticks from cats (Table 4), which did not differ significantly compared with dogs (*χ*^2^ test, *χ*^2^ < 0.001, *df* = 1, *P* = 1). By Sanger sequencing, *B. garinii* was identified in 20/24 (83.3%) and *B. bavariensis* in 1/24 (4.2%) of *B. garinii*/*B. bavariensis*-positive ticks from cats, while *B. burgdorferi* s.s. was identified in 11/17 (64.7%) of *B. burgdorferi* s.s./*B. carolinensis*-positive ticks. The nine remaining samples with ambiguous RLB results could not be further differentiated owing to coinfections.

### Prevalence and copy numbers of *A. phagocytophilum* and *Borrelia* spp. in relation to tick engorgement status

**Fig. 2:**
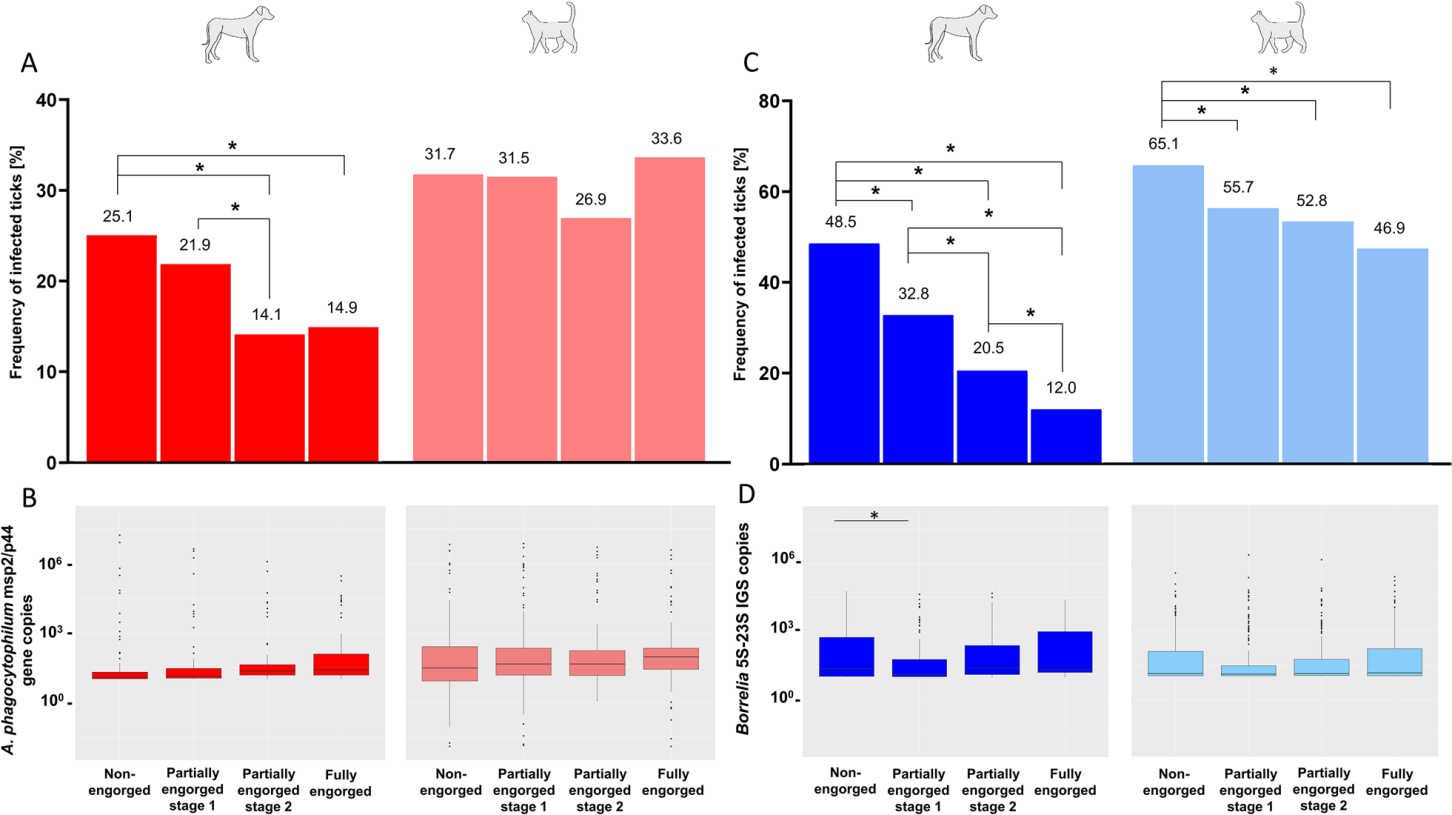
Differences in the frequency of ticks infected with *A. phagocytophilum* (**A**) and *Borrelia* spp. (**C**) as well as *A. phagocytophilum* msp2/p44 gene (**B**) and *Borrelia* 5S–23S IGS copies (**D**) per stage of engorgement in ticks from dogs (red/royal blue) and cats (light red/light blue). Boxes extend from the 25th to the 75th percentile, with a line at the median and whiskers extending to 1.5 the interquartile range. Significant differences (*P* < 0.05) are indicated by an asterisk

In ticks collected from dogs, the highest prevalence of *A. phagocytophilum* as well as *Borrelia* spp. was measured in non-engorged specimens, decreasing significantly with increasing time of engorgement (Fig. 2). Regarding *A. phagocytophilum*, the prevalence amounted to 25.1% (94/375) in non-engorged ticks but only to 14.9% (56/375) in fully engorged ticks, with a significant difference between non-engorged ticks and partially engorged ticks from stage 2 or fully engorged specimens, as well as between partially engorged ticks from stage 1 and stage 2 (Table 2). The median *A. phagocytophilum* copy numbers varied between 6.96 × 10^1^ in non-engorged and 1.80 × 10^2^ in fully engorged ticks, but the increase with increasing engorgement time was not statistically significant after *P*-value correction (Kruskal–Wallis test, *χ*^2^ = 8.52, *df* = 3, *P* 0.036; Fig. 2, Dunn’s post hoc tests, *P* = 0.077 between non-engorged and fully engorged ticks).

**Fig. 3:**
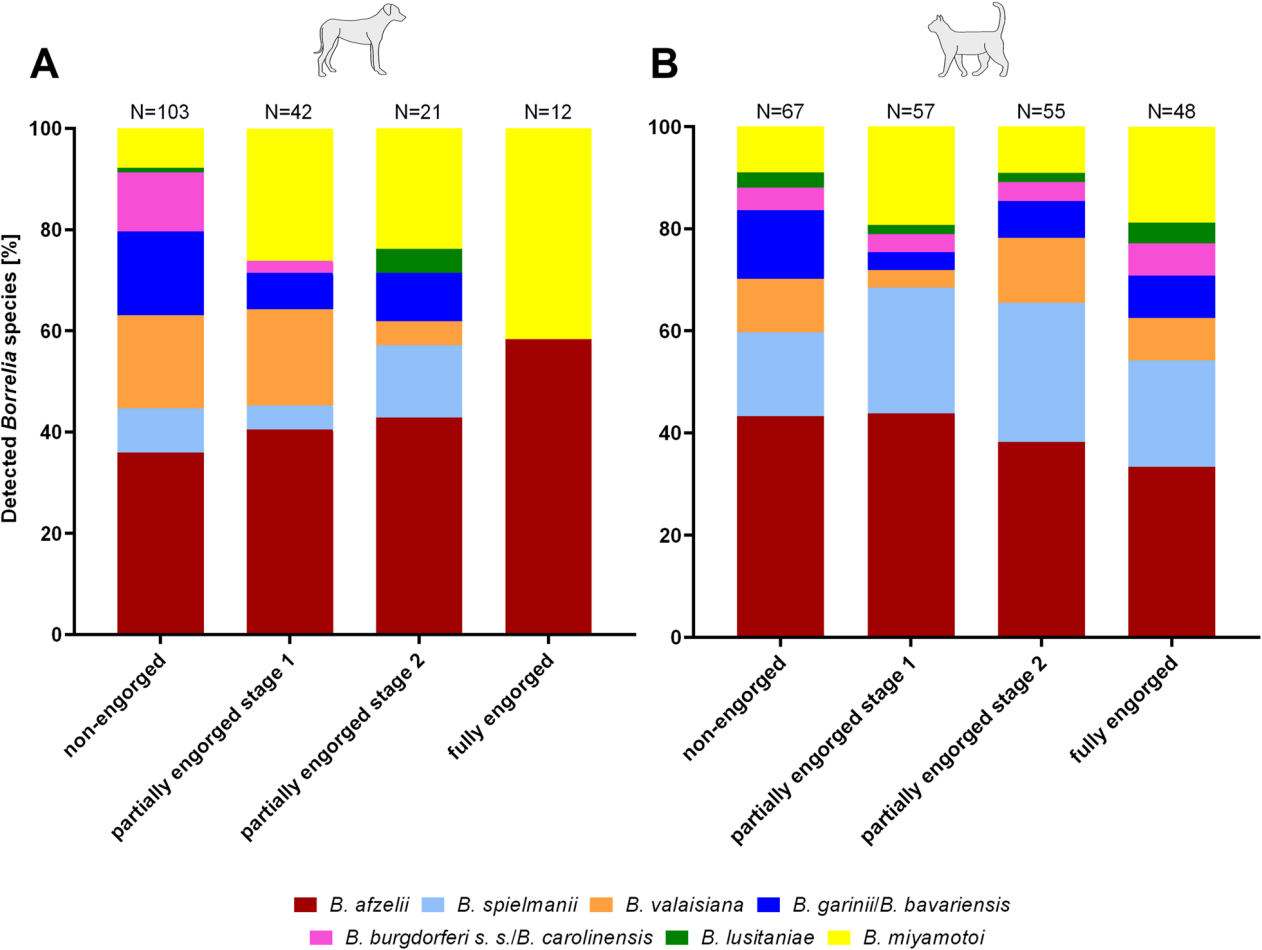
*Borrelia* species distribution among successfully differentiated ticks from dogs (**A**) and cats (**B**) per stage of engorgement. Note the different sample size of positive ticks for each engorgement category indicated above the bars

Concerning *Borrelia* spp., with 48.5% (182/375) of the non-engorged ticks from dogs being qPCR positive but only 12.0% (45/375) of the fully engorged ticks, all stages of engorgement showed significantly different prevalence values compared with non-engorged ticks and the other engorgement stages (Table 2). Seven different *Borrelia* species were detected in non-engorged ticks, six each in partially engorged ticks of phase 1 and phase 2, and two, precisely *B. afzelii* and *B. miyamotoi*, in fully engorged specimens (Fig. 3). A significant difference in prevalence between the engorgement categories was only detected with regard to *B. miyamotoi* (Fisher’s exact test, Bonferroni–Holm-corrected* P* = 0.007). Further, median *Borrelia* spp. copy numbers varied between 5.68 × 10^2^ copies in non-engorged and 3.75 × 10^2^ copies in fully engorged ticks (Fig. 2). Additionally, a significant influence of the time of engorgement on the *Borrelia* copy numbers was detected (Kruskal–Wallis test, *χ*^2^ = 13.54, *df* = 3, *P* = 0.004), with significantly higher copy numbers in non-engorged specimens compared with partially engorged ticks of stage 1 (Dunn’s post hoc test, *P* = 0.003).

In ticks from cats, no significant difference in *A. phagocytophilum* prevalence between the stages of engorgement was noted, with a prevalence of 33.6% (126/375) in fully engorged ticks and 31.7% (119/375) in non-engorged specimens (Table 2). Furthermore, no significant influence of the time of engorgement on the *Anaplasma* copy numbers was detected (Kruskal–Wallis test, *χ*^2^ = 3.44, *df* = 3, *P* = 0.328) with a median of 8.85 × 10^1^ copies in non-engorged and 1.52 × 10^2^ copies in fully engorged ticks (Fig. 2).

Concerning *Borrelia* spp., a significantly higher prevalence was detected in non-engorged (244/375; 65.1%) than in partially (stage 1: 209/375; 55.7%; stage 2: 198/375; 52.8%) and fully engorged ticks from cats (176/375; 46.9%) (Fig. 2; Table 2). However, no changes in *Borrelia* species distribution over the time of engorgement were noted in ticks from cats (Fig. 3). With regard to *Borrelia* copy numbers, with medians between 1.11 × 10^2^ copies in non-engorged and 6.50 × 10^1^ copies in fully engorged ticks, no significant influence of engorgement status was detected (Kruskal–Wallis test, *χ*^2^ = 4.19, *df* = 3, *P* = 0.242; Fig. 2).

### Pathogen prevalence in single and multiple infested dogs and cats

In total, ticks from 535 single infested dogs and 371 cats were selected for pathogen detection, as well as a total of 965 ticks from 474 multiple infested dogs, and 1129 ticks from 527 multiple infested cats.

Concerning *A. phagocytophilum*, 17.4% (93/535) of the single infested dogs harbored infected ticks, and 24.0% (89/371) of the single infested cats. Regarding multiple infested animals, overall 32.1% (152/474) of the dogs harbored at least one positive tick compared with 51.8% (273/527) of the multiple infested cats (Table 5).

**Table 5:** Prevalence of *A. phagocytophilum* and *Borrelia* spp. in female *I. ricinus* from single and multiple infested dogs and cats

	No. of dogs harboring positive ticks %	No. of positive ticks from dogs %	No. of cats harboring positive ticks %	No. of positive ticks from cats %
*A. phagocytophilum*				
Single infestation	93/535 (17.4)	93/535 (17.4)	89/371 (24.0)	89/371 (24.0)
Multiple infestation	152/474 (32.1)	192/965 (19.9)	273/527 (51.8)	375/1129 (33.2%)
Total	245/1009 (24.3)	285/1500 (19.0)	362/898 (40.3)	464/1500 (30.9)
*Borrelia* sp.				
Single infestation	130/535 (24.3)	130/535 (24.3)	184/371 (49.6)	184/371 (49.6)
Multiple infestation	243/474 (51.3)	297/965 (30.8)	417/527 (79.1)	643/1129 (57.0)
Total	373/1009 (37.0)	427/1500 (28.5)	601/898 (66.9)	827/1500 (55.1)

For *Borrelia* spp., 24.3% (130/535) of the single infested dogs carried infected ticks and 49.6% (184/371) of the single infested cats. Concerning multiple infestations, 51.3% (243/474) of the dogs harbored at least one positive tick, and 79.1% (417/527) of the cats (Table 5). There was no significant difference in prevalence of *A. phagocytophilum* nor *Borrelia* spp. between ticks from single and multiple infestations of dogs, but both occurred significantly more often in ticks from multiple than single infested cats (Table 2).

Considering only cases of multiple infested animals, the number of actually examined ticks varied from one to five. From 450 dogs, more than one tick was examined, of which 20.0% of the examined ticks (188/941) from 148 dogs were *A. phagocytophilum* positive. Concerning cats, 492 animals contributed more than one tick to pathogen detection, of which 364/1.096 (33.2%) specimens from 262 cats were *A. phagocytophilum* positive. The distribution of dogs infested with qPCR-positive ticks was as follows: In 73.7% (109/148) of cases, only a single tick was *A. phagocytophilum* positive, while two ticks were positive in 25.7% (38/148) of cases and three ticks in only one case (0.7%). Regarding cats, 66.0% (173/262) contributed a single, 30.5% (80/262) contributed two, and 3.4% (9/262) contributed three or more *A. phagocytophilum*-positive ticks (Table 6).

**Table 6:** Distribution of animals from which multiple female *I. ricinus* were examined according to the number of positive ticks per host. The upper part of the table includes only engorged ticks, while all ticks are included in the lower part

	Only engorged ticks										
	No. of positive ticks on examined dogs					No. of positive ticks on examined cats					
	None	One	Two	Three	Four	None	One	Two	Three	Four	Five
*A. phagocytophilum*											
Hosts with two examined ticks	181/244	47/244	16/244			131/267	102/267	34/267			
Hosts with three examined ticks	9/17	5/17	2/17	1/17		11/33	9/33	9/33	4/33		
Hosts with four examined ticks	–	–	–	–	–	1/6	1/6	2/6	0/6	2/6	
*Borrelia* spp.											
Hosts with two examined ticks	147/244	82/244	15/244			61/267	116/267	90/267			
Hosts with three examined ticks	10/17	6/17	1/17	0/17		5/33	9/33	14/33	5/33		
Hosts with four examined ticks	–	–	–	–	–	0/6	4/6	1/6	1/6	0/6	

	All ticks										
	No. of positive ticks on examined dogs					No. of positive ticks on examined cats					
	None	One	Two	Three	Four	None	One	Two	Three	Four	Five
*A. phagocytophilum*											
Hosts with two examined ticks	280/412	99/412	33/412			193/391	143/391	55/391			
Hosts with three examined ticks	20/35	10/35	4/35	1/35		34/91	28/91	23/91	6/91		
Hosts with four examined ticks	2/3	0/3	1/3	0/3	0/3	2/7	2/7	2/7	1/7	0/7	
Hosts with five examined ticks	–	–	–	–	–	1/3	0/3	0/3	0/3	0/3	2/3
*Borrelia* spp.											
Hosts with two examined ticks	202/412	166/412	44/412			78/391	169/391	144/391			
Hosts with three examined ticks	15/35	11/35	8/35	1/35		12/91	21/91	38/91	20/91		
Hosts with four examined ticks	2/3	1/3	0/3	0/3	0/3	0/7	5/7	1/7	1/7	0/7	
Hosts with five examined ticks	–	–	–	–	–	1/3	1/3	0/3	1/3	0/3	0/3

Concerning *Borrelia* spp. among ticks from animals that contributed more than one examined tick, 285/941 (30.3%) infected ticks were collected from 231 dogs, compared with 627/1096 (57.2%) positive specimens from 401 cats. From most dogs, only a single tick was positive (178/231; 77.1%), while in 22.5% (52/231) of cases two ticks were infected and three ticks in only one case (0.4%). Concerning cats, 48.9% (196/401) contributed a single *Borrelia* infected tick, 45.6% (183/401) contributed two, and 5.5% (22/401) contributed three infected ticks. By excluding non-engorged specimens, which had not yet had a chance of becoming infected by the current bloodmeal, no change within this distribution pattern was visible (Table 6).

## Discussion

Owing to climatic and environmental changes over the last decades, the geographical and seasonal expansion of ticks toward year-round activity translates to an increased overall risk of infestation and thus infection with tick-associated pathogens such as *A. phagocytophilum* and *Borrelia* spp., endangering human and animal health. A tick submission study from Germany revealed a long average time of engorgement of female *I. ricinus* ticks on dogs (78.8 h) and cats (82.7 h), illustrating the high risk of pathogen transmission [30]. Especially regarding cats, the current risk of infection with TBDs is probably underestimated, as a rising number of feline TBD case reports illustrates [9, 44].

In previous studies on questing female *I. ricinus* collected from vegetation in Germany, *A. phagocytophilum* prevalences between 1.0% [11] and 11.6% [45] were detected, whereas a comparably high prevalence of 40.5% was reported from Denmark [12]. In previous studies examining ticks from dogs and cats, between 0.1% female *I. ricinus* from the UK [15], 3.5% from Finland [20], and 22.3% from Poland [16] were found to be *A. phagocytophilum* positive. In the present study examining exclusively female *I. ricinus* ticks, the prevalence in ticks from dogs was quite high compared with these values, with every fifth tick positive, and even higher in ticks from cats with almost every third tick positive. The quite high *A. phagocytophilum* prevalence might additionally be influenced by the significant positive association with *Borrelia* spp. infection status, and vice versa. Enhanced acquisition of both organisms by larval ticks from coinfected mice has been shown, related to higher bacterial burdens [46]. Moreover, coinfections with *B. burgdorferi* s.l. and *A. phagocytophilum* were frequently detected in *I. ricinus* collected from European hedgehogs [47].

Concerning *Borrelia* spp., most studies from Germany and neighboring countries have determined a prevalence around 35.0% in questing, non-engorged female *I. ricinus* [3, 17, 29, 40, 48, 49], while the prevalence reached from 1.0% in a study examining adult *I. ricinus* ticks from dogs sampled in four European countries [26], over 10.5% in a recently published study from Finland [20], to 44.0% in adult engorged *I. ricinus* ticks from wild and domestic animals in the Ukraine [24]. Therefore, the prevalence of 28.5% in female *I. ricinus* ticks collected from dogs in the present study is similar to that in questing ticks, but considerably lower than the detected infection rate in ticks from cats, with more than every second tick positive for *Borrelia* species.

The fact that the seroprevalence of anti-*A. phagocytophilum* and anti-*Borrelia* spp. antibodies in dogs varies enormously in Europe between 1.1% and 56.5% as reviewed by [50], or 1.1% and 17.9% as reviewed by [51], additionally shows that regional investigations can hardly be extrapolated, underlining the importance of nationwide surveys. In the present study, however, the investigated pathogens were detected more or less evenly across Germany.

To evaluate whether the detected prevalence might be influenced by the ticks’ engorgement status, spiking trials were performed to exclude possible PCR inhibition due to the bloodmeal, although a DNA isolation kit specifically designed to inactivate PCR inhibitors from blood and other body fluids had already been used. The spiking trials showed no influence of the stage of engorgement, but inhibitors actually did interfere with the qPCR process as false-negative results were obtained in non-engorged as well as engorged ticks when using 10 µl and 5 µl template volume. Interestingly, copy numbers were mainly not affected, being comparable in almost all samples with positive results. Thus, the inhibition does not seem to skew qPCR quantification, but suppresses the detection in general in certain cases similar to a “yes or no” answer. The *Borrelia* qPCR seemed to be more susceptible to inhibition than the *A. phagocytophilum* qPCR. Differential susceptibility of different PCR assays to inhibitors has been reported previously [52]. Of note, inhibition was only observed when using isolated DNA from host-detached ticks, but not when using DNA from questing ticks as template, suggesting that the initiation of feeding activates or introduces an inhibitor in the tick. If this inhibitor has a biological function, e.g., in the context of an immunomodulation at the location of the tick bite, needs to be investigated further. Therefore, studies on host-detached ticks may be prone to false-negative results at certain template quantities, which should be carefully evaluated in future studies. In the present study, the qPCR was repeated with reduced template volume (2 µl) for every negative tick afterwards, thereby detecting more *A. phagocytophilum*- and *Borrelia*-infected ticks in every engorgement category, but the general pattern over the stages of engorgement nevertheless remained as initially detected.

Concerning the overall prevalence of the pathogens detected, the significant differences that were determined between ticks from dogs and cats may be related to the different outdoor behavior and the resulting degree of habitat overlap with or contact to TBP reservoir hosts. Contact of cats with hedgehogs and other small mammals, acting as reservoir hosts for *A. phagocytophilum* and a variety of *Borrelia* species [53–55], may be provoked by their unrestricted hunting drive. This is further supported by a veterinary submission study showing differences in the distribution of tick species infesting dogs and cats, with 5.0% of the collected ticks identified as the hedgehog tick *Ixodes hexagonus* representing the second most frequently detected tick species on cats, while in dogs only 1.6% of the collected ticks were identified as *I. hexagonus* [30]. Further, infected ticks might show a stronger affinity to cats than dogs, however, this speculation requires further investigations.

Differences between ticks from dogs and cats were also apparent regarding the *Borrelia* species distribution, further underlining the hypothesis that both host species have a different probability of acquiring ticks that previously fed on certain reservoir hosts. In ticks from both host species, *B. afzelii* was detected most frequently, in line with former studies on questing and/or host-associated ticks [3, 27, 40, 48, 56]. However, *B. miyamotoi* and *B. valaisiana* were more frequently detected in ticks from dogs than *B. garinii*, which was the second most frequently detected species in the above-mentioned previous studies, whereas in ticks from cats, *B. spielmanii* and *B. miyamotoi* were more frequent than *B. garinii*. Further, *B. valaisiana*, which is associated with birds or lizards as reservoir hosts [57], was detected significantly more frequently in ticks from dogs than cats, while cats harbored significantly more ticks infected with *B. spielmanii*, whose main reservoir hosts are rodents but which is also found in hedgehogs [54, 58].

While in ticks from dogs, the *Borrelia* species was successfully determined in more than every third tick by RLB, the differentiation rate was lower for ticks from cats, with only every fourth tick successfully examined. This is most likely related to the fact that 430/827 *Borrelia*-positive ticks from cats contained only very low copy numbers (≤ 10^1^ copies), hampering detection by RLB, which is less sensitive than qPCR. For both host species, the overall differentiation success rate was comparable to those from previous studies (31.6%-58.0%) examining questing [3, 40, 49] and host-associated ticks [17].

To examine whether dogs and cats might act as reservoir hosts themselves, infecting the infesting ticks, or whether tick-to-tick transmission by cofeeding might take place during the bloodmeal, pathogen prevalence between ticks from single and multiple infestations as well as between different stages of engorgement was compared. Concerning cats, a multiple infestation type significantly raised the odds of a tick to be pathogen-positive compared with single infestations, while in dogs multiple infestations had no significant influence on the detected pathogen prevalence. Further, in those cases where more than one tick was examined per host, most dogs carried just a single infected tick, while only a quarter harbored more than two to three infected ticks at the same time. In cats that contributed multiple ticks to the analysis, the proportion of animals with more than one infected tick was approximately one-third for *A. phagocytophilum* and one-half for *Borrelia* spp., however, this distribution remained the same when excluding non-engorged specimens, which have had little or no chance yet to become infected by the current bloodmeal. Thus, a reservoir function of the cat itself is unlikely, but the data rather suggest a higher pathogen prevalence in ticks from the cats’ environment than in the natural habitat of dogs. Further, the attachment sites of the individual ticks argue against a potential influence of cofeeding, as in 55.9% of cases with two or more positive ticks per animal, those ticks were detected on separate body parts (data not shown).

Moreover, a decreasing rather than an increasing frequency of infected ticks was noted with increasing engorgement duration, except for *A. phagocytophilum* in ticks from cats, where no significant differences were visible at all. One of the main hypotheses for an increasing *Borrelia* prevalence over the time of engorgement is feeding-induced multiplication of spirochetes [59]. According to that, a study by Michalski et al. [17] showed a higher prevalence of *Borrelia* spp. and *A. phagocytophilum* in engorged females compared with non-engorged specimens collected from dogs, although the sample sizes of examined non-engorged ticks were very small with 21 specimens for *Borrelia* spp. and 11 for *A. phagocytophilum* compared with a higher sample size of 436 and 402 engorged specimens, respectively, in that study. In contrast, a study from the Netherlands showed a decreasing *Borrelia* prevalence from non-engorged (11.8%; 10/85) to partially engorged ticks (1.9%; 1/53) collected from dogs [60], comparable to the findings of the present study. Other studies also described a decreasing prevalence with increasing time of engorgement [29, 61]. This might be related to an early transmission of *Borrelia* spirochetes, an elimination by defecation or a destruction during the time of transfer from midgut to salivary glands [60, 62]. Further reasons might be a dilution effect due to the engorged blood or complement-mediated *Borrelia* clearance, as has been described for ruminant blood [63]. However, complement-mediated clearance seems less likely here, as most members of the *B. burgdorferi* s.l. complex are resistant to serum complement of dogs and cats, except for an intermediate susceptibility of *B. garinii* and *B. lusitaniae* as reviewed by [64]. Further, there are strain-specific differences in serum susceptibility, and a German *B. garinii* strain (Pbi) showed a high resistance to dog serum in another study [65]. Moreover, in adult female ticks removed from humans, both *B. garinii* and *B. afzelii* bacterial load decreased with increasing engorgement duration, with a stronger decrease of *B. afzelii* [66], despite the fact that *B. garinii* but not *B. afzelii* is considered susceptible to human complement [64], indicating that immune-mediated clearance probably only played a minor role for the decrease in bacterial load. The *Borrelia* copy numbers in fully engorged ticks in the present study did not differ from those in non-engorged ticks, but a significant decrease of copy numbers was observed between non-engorged ticks from dogs and stage 1 partially engorged specimens. This may indicate efficient transmission of *Borrelia* spirochetes to dogs within the early phase of the bloodmeal, supported by the biggest drop of prevalence between these two engorgement stages. The result is comparable to the decrease of *B. afzelii* and *B. garinii* load in ticks having fed for more than 36 h on humans, which the authors linked to possible pathogen transmission [66]. Further, neither multiplication of the *Borrelia* spp. in the midgut nor a dilution effect are evident from the present data, as no significant increase nor decrease of the detected copy numbers occurred after this initial decrease. As a cautionary note, it should be kept in mind that the investigated target is a multi-copy gene in *B. burgdorferi* s.l. but a single-copy gene in *B. miyamotoi*, indicating that the copy numbers are not necessarily linearly related to bacterial load.

Similar to the picture in ticks from dogs, the frequency of *Borrelia*-infected ticks decreased significantly, but not as sharply, in ticks from cats. Further, in ticks from cats, no significant decrease of the detected copy numbers was evident, possibly indicating a less efficient transmission of *Borrelia* spirochetes compared with the findings in ticks from dogs, which might go hand in hand with minor clinical symptoms in cats.

With regard to *A. phagocytophilum*, only Michalski et al. [17] compared non-engorged and engorged ticks from dogs. Further studies have been performed on ticks from ruminants, such as roe deer, showing a distinct *A. phagocytophilum* prevalence increase with increasing time of engorgement [37], which, however, might be related to the reservoir function of wild ruminants as reviewed in [67]. In the present study, a distinct decrease of the *A. phagocytophilum* prevalence between stage 1 (24–72 h) and stage 2 (72–144 h) partially engorged ticks from dogs was observed, which could possibly indicate the time window when most transmission takes place. Even if the transmission of *A. phagocytophilum* can already occur after 6 h of feeding, although seldomly resulting in a sufficient infection [68], it is commonly agreed that the onset of transmission occurs around 36 h after the beginning of the feeding process [69]. Nevertheless, the *A. phagocytophilum* copy numbers were not significantly affected by increasing time of engorgement in ticks from dogs nor in ticks from cats. Only in ticks from dogs, a slight increase might indicate that the pathogen is replicating.

Cats also did not show a decline in *A. phagocytophilum* prevalence, which suggests different transmission kinetics related to the different host species. In general, dogs are regarded as more suitable *A. phagocytophilum* hosts compared with cats as reviewed in [67]. Further studies are necessary to establish the cause of the decreasing prevalence in ticks of dogs with certainty as well as why this decrease was not or not as apparent in ticks from cats and to explain the observed differences between dogs and cats.

## Conclusions

The high prevalence of *A. phagocytophilum* and *Borrelia* spp. detected in ticks from dogs and cats in this study underlines the high risk of pathogen transmission and thus the need for efficient tick protection via licensed acaricides, as e.g. recommended by the European Scientific Counsel Companion Animal Parasites (ESCCAP), to protect the health of dogs and cats with regard to clinical cases of anaplasmosis or borreliosis. Even if the relevance of *Borrelia* spp. in veterinary medicine is lower than in human medicine, ticks that are introduced into the human environment with companion animals and then wiped off may represent a risk for humans. Thus, tick protection of dogs and cats is also of public health relevance within a One Health context. The significant differences between dogs and cats concerning the overall pathogen prevalence, coinfections, and *Borrelia* species distribution are indicative of behavioral differences resulting in varying degrees of contact or habitat overlap with different TBP reservoir hosts. Decreasing prevalence of *A. phagocytophilum* as well as *Borrelia* spp. in ticks from dogs with increasing time of engorgement may indicate successful transmission, while cat blood might negatively affect the efficiency of *A. phagocytophilum* transmission. This could be a further explanation for the low number of clinical cases and/or less pronounced clinical symptoms in cats. However, further studies are needed to unravel the differences between dogs and cats in this regard.

## Data Availability

Data supporting reported results is contained within the article.

## Abbreviations

AIC: Akaike information criterion
Ct: Cycle threshold
ESCCAP: European Scientific Counsel Companion Animal Parasites
GLMM: Generalized linear mixed model
RLB: Reverse line blot
SD: Standard deviation
TBD: Tick-borne diseases
TBP: Tick-borne pathogens
qPCR: Quantitative real-time polymerase chain reaction
